# Retraction: Genome-Wide Identification and Expression Analysis of WRKY Gene Family in *Capsicum annuum* L.

**DOI:** 10.3389/fpls.2016.01727

**Published:** 2016-11-04

**Authors:** Frontiers in Plant Science

The journal retracts the 23 February 2016 article cited above. Following concerns regarding the originality of the article, Frontiers conducted an investigation. The investigation determined an unacceptably high level of similarity with an article published by Diao et al. (2015) in *Acta Horticulturae Sinica*. This retraction was approved by the Field Chief Editor and the Specialty Chief Editor of *Frontiers in Plant Science*. The authors concur with the retraction and sincerely regret any inconvenience this may have caused to the reviewers, editors, and readers of Frontiers in Plant Science.
